# Case report of intracranial large vessel occlusion in glioblastoma multiforme patient after radiation therapy

**DOI:** 10.1097/MD.0000000000032682

**Published:** 2023-01-13

**Authors:** Yang Chien-Tung, Chun-Chung Chen

**Affiliations:** a Neurosurgical department, China Medical University Hospital, Taichung, Taiwan; b Department of Surgery, College of Medicine, China Medical University, Taichung, Taiwan.

**Keywords:** case report, extracranial-intracranial bypass surgery, GBM, radiation vasculopathy

## Abstract

Vasculopathy of the large arteries is a prominent complication of radiotherapy. Radiation-induced cerebral vasculopathy can cause arterial stenosis/occlusion, cerebral hemorrhage, and aneurysm formation. We report a cases of glioblastoma multiforme (GBM) with occlusive radiation vasculopathy (ORV).

**Case presentation:**

This 28-year-old patient who suffered from GBM had surgery for cytoreduction and received postoperative CCRT. We adopted the radiotherapy and oncology group radiation guideline. This patient had cerebrovascular accident episodes without any known risk. Therefore, ORV was highly suspected and vascular stenosis was confirmed using magnetic resonance angiography (MRA) or digital subtraction angiography. Extracranial-intracranial bypass was performed and patency was confirmed. The patient had not suffered from recurrent symptoms of transient ischemic attack or ischemic stroke for 1.5 years.

**Discussion:**

This is the first article to report bypass surgery for GBM patients. Although the median survival rate of GBM is approximately 15 months, the short survival time may be sufficient for occlusive vasculopathy to occur. Regular follow-up magnetic resonance imaging assessments are recommended, as is MRA as a screening tool for the early diagnosis of ORV.

The Stenting versus Aggressive Medical Management for Preventing Recurrent Stroke in Intracranial Stenosis (SAMMPRIS) trial focused on atherosclerotic intracranial arterial stenosis, revealing that aggressive medical management was superior to stenting for secondary stroke prevention; however, it did not mention radiation-induced vasculopathy. Bypass surgery has yielded some positive outcomes. In the absence of contraindications, antiplatelet or anticoagulation agents could be added, and bypass surgery could be performed because there was no stent in the distal intracranial arteries.

**Conclusion:**

MRA is a potential screening tool for ORV in GBM patients and bypass surgery could be performed to improve brain perfusion. Bypass surgery could help patient with occlusive radiation vasculopathy

## 1. Introduction

Radiation therapy (RT) is a main treatment for glioblastoma multiforme (GBM) patients. The standard treatment for GBM comprises maximal safe cytoreduction resection followed by combined chemoradiotherapy. Vasculopathy of the large arteries is a prominent complication post-RT and is established in the fields of pediatric brain tumor and head and neck cancer. The latency of radiation vasculopathy diagnosis ranges from 2 to 25 years, with a peak incidence at 3 years.^[1–4]^ Radiation-induced cerebral vasculopathy is an umbrella term for arterial stenosis, occlusion, cerebral hemorrhage, aneurysm formation, and cavernomas.^[5]^ It can lead to clinical manifestations such as ischemic or hemorrhagic stroke and delayed cognitive impairment.^[6]^

The incidence of cerebral artery stenosis is approximately 8.6% in patients undergoing RT for head and neck cancer and 6.7% in patients who had received proton RT in childhood.^[7,8]^ Cerebral artery stenosis or occlusion after RT is relatively uncommon in glioma patients; however, reports are increasing as long-term survival increases.

Here, we discuss a certain type of vascular radiculopathy, occlusive radiation vasculopathy (ORV), in a GBM patient. ORV occurred within a year post-RT and was treated with extracranial-intracranial bypass surgery.

## 2. Case report

**Figure 1.**
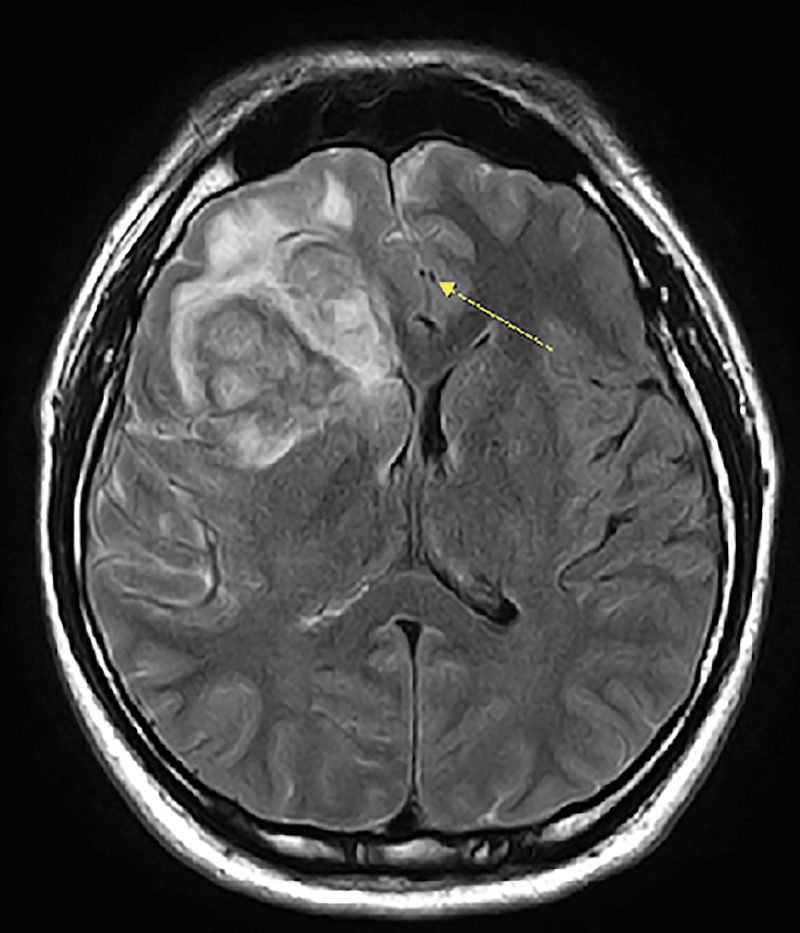
MRI T2 FLAIR showing the tumor location. The PTV definitely includes the bilateral anterior communicating artery (yellow arrow). MRI = magnetic resonance imaging.

**Figure 2.**
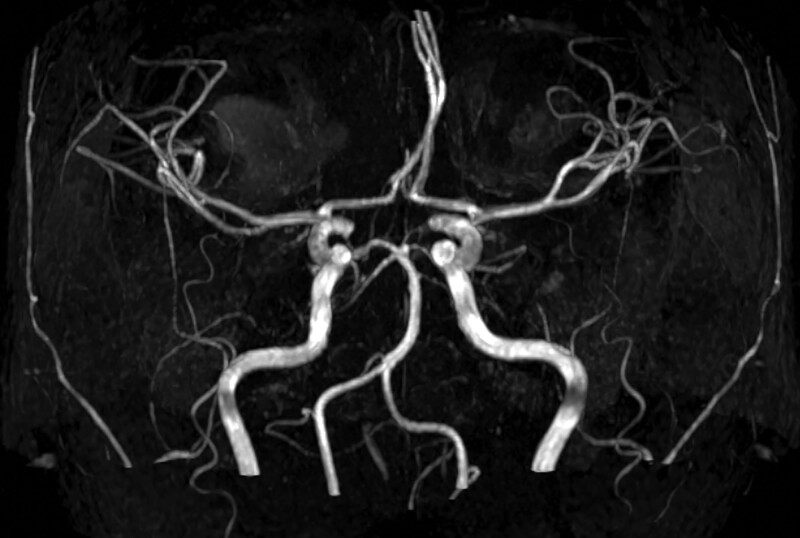
MRA indicates no ACA narrowing. MRA = magnetic resonance angiography.

The patient was a 28-year-old man without cerebrovascular accident risk factors such as arrhythmia, overweight, hyperlipidemia, DM, and hypertension. He was diagnosed with right frontal GBM (IDH wild-type) (Figs. 1 and 2) and underwent surgery on April 17, 2020, followed by CCRT. We adopted the Radiotherapy and Oncology Group (RTOG) guideline.^[9]^ The RTOG recommendation is composed of 2 phases, first 46 Gy then followed by 14 Gy boost. The first phase planning target volume (PTV) is surgical resection cavity with residual enhancing tumor (postcontrast T1 weighted magnetic resonance imaging [MRI]) and surrounding edema (hyperintensity on T2 or FLAIR MRI) plus margin; while the second phase PTV is surgical resection cavity with residual enhancing tumor (postcontrast T1 weighted MRI) plus margin. The radiation lasted 6 weeks, from May 14 to June 14, 2020. He underwent secondary surgery for recurrent GBM resection on July 29, 2020.

**Figure 3.**
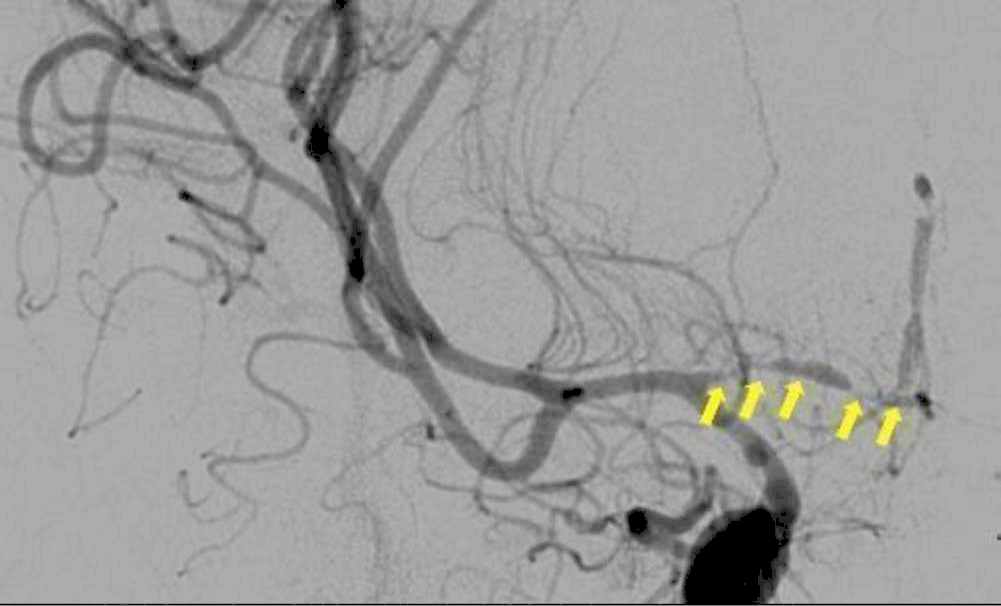
Right ICA angiography reveals focal narrowing with dilatation (yellow arrow) from right A1 to A2 of ACA, occlusion at distal A2 ACA.

**Figure 4.**
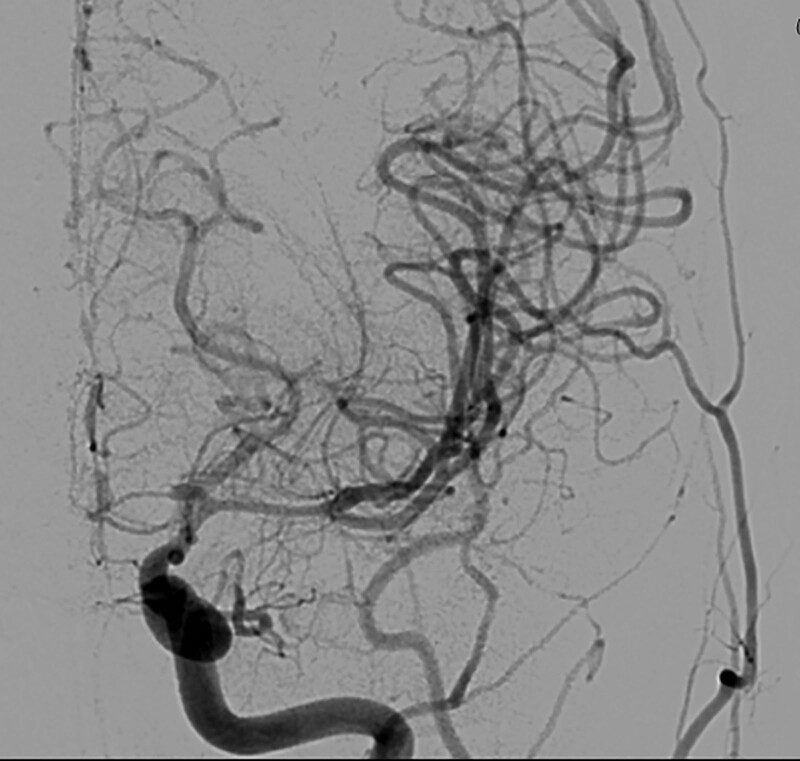
Left CCA angiography reveals occlusion of the left A1 ACA.

**Figure 5.**
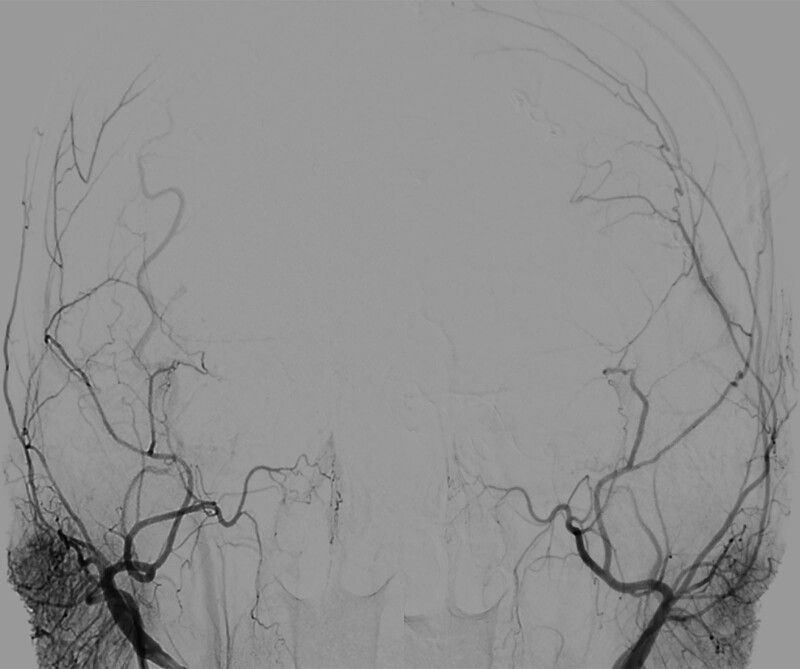
Patency of ECIC.

However, on March 03, 2021, he suffered sudden hemiparesis; MRI revealed acute infarction on bilateral ACA territory and left MCA territory. Magnetic resonance angiography (MRA) and digital subtraction angiography revealed the string sign in the bilateral A1 segments as well as occlusion and left MCA stenosis (Figs. 3 and 4). In April 2020, MRA indicated no stenosis of the intracranial vessels before GBM treatment, especially the bilateral ACA. According to the tumor location, there was no chance for the operator to injure the contralateral ACA branch. However, the planning target volume (PTV) contained the contralateral ACA. In approximately 9 months, he developed symptomatic radiation vasculopathy. We started administering 100 mg of aspirin every day after that. Left side direct bypass (superficial temporal artery [STA] and MCA 4th segment [M4] anastomosis) with indirect bypass eencephaloduroarteriosynangiosis and right STA-radial artery-ACA (A3) bypass were performed. Postoperatively, he exhibited progressive recovery from hemiparesis and no stroke attacks, and follow-up digital subtraction angiography confirmed the patency of the bypass (Fig. 5). The patient is still alive (2022 November) under immunotherapy and he had not suffered from recurrent symptoms of transient ischemic attack or ischemic stroke for 1.5 years.

## 3. Discussion

We reported a case of GBM patients with ORV and to the best of our research this is the first case regarding this issue. With a total 60 Gy dose, the interval between RT and occlusive vasculopathy was < a year. Although the median survival length of GBM is approximately 15 months,^[10]^ it may be sufficient for occlusive vasculopathy to develop. Because ORV can affect GBM patients, early detection is required. According to the NCCN guidelines for newly diagnosed GBM, patients should be followed closely with serial brain MRI scans (at 2–8 weeks post-radiation, then every 2–4 months for 3 years, and then every 3–6 months indefinitely) after treatment completion.^[11]^ Since regular MRI is recommended, we suggest MRA as a screening tool for the early diagnosis of ORV, especially for vessels located near the PTV.

Radiation vasculopathy is a well-described late effect in pediatric brain tumors and head and neck squamous cell carcinoma (HNSCC). In addition, RT is also a primary therapy used to treat brain tumors. Radiation-induced cerebral vasculopathy has been reported after RT for pediatric brain tumors, most commonly gliomas and medulloblastomas.^[12]^ The potential adverse effects of RT on the brain include radiation necrosis, atrophy, gliosis, telangiectasia, cavernous malformations, and the development of large-vessel cerebral arteriopathies, including moyamoya syndrome.^[13–17]^ Vascular injury may develop soon or decades post-RT.^[18]^ The mechanism of radiation-induced vasculopathy is unclear, but inflammatory changes lead to necrosis, fibrosis, hyalinization and occlusion of vessels.^[19,20]^

Although radiation-induced vasculopathy can be diagnosed histopathologically, the diagnosis is primarily based on radiologic evidence of arterial stenosis or the occlusion of a previously normal large intracranial vessel. The incidence of cerebral artery stenosis is approximately 8.6% in patients undergoing RT for head and neck cancer and 6.7% in patients who received proton RT in childhood.^[21,22]^ Campen et al reported a 100-fold increase in the risk of transient ischemic attack or stroke compared to that in the general population, a median time of 4.9 years from the initial radiation to stroke and an increased risk of stroke in patients who received RT to the circle of Willis.^[23]^ Similarly, El-Fayech et al demonstrated that a radiation dose of 10 Gy or more to the circle of Willis was associated with a cumulative stroke incidence of 11.3% compared with an expected incidence of 1% in the general population.^[24]^ In a Childhood Cancer Survivor Study cohort for children who received > 50 Gy cranial radiation, the cumulative incidence of stroke increased from 1.1% at 10 years after diagnosis to 12% after 30 years.^[25]^ Piotr et al reported 26 cases of head & neck squamous cell carcinoma with a mean irradiation dose of 62 Gy (58–72 Gy); the mean interval from RT to symptomatic stenosis was 3 to 7 years and the shortest time was < 2 years.^[26]^ Dorresteijn et al reported a median interval of 10 years for stroke in head and neck cancer patients irradiated with 60 to 70 Gy.^[27]^ In both fields, some studies suggest that vasculopathy screening is required.^[7,8]^

Currently, no guidelines are available for managing stroke secondary to radiation vasculopathy. The Stenting versus Aggressive Medical Management for Preventing Recurrent Stroke in Intracranial Stenosis (SAMMPRIS) trial, which focused on atherosclerotic intracranial arterial stenosis, revealed that aggressive medical management was superior to stenting for secondary stroke prevention. However, it did not mention radiation-induced vasculopathy.^[28]^ In the SAMMPRIS trial, aggressive medical treatment included antiplatelet drugs (clopidogrel and aspirin) and controlling the lipid profile, blood sugar levels, and blood pressure. However, the same strategy may not be suitable for radiation vasculopathy owing to differing pathogenesis.^[29–31]^ Sometimes, patients are young without any atherosclerosis risk factors. In this trial, the stent was placed in the ICA, MCA, vertebral artery (VA), and basilar artery (BA). Lesion sites involving more distal branches may hinder stent placement. In another study, Gao et al reported that the intracranial stent can substantially reduce the 1-month stroke and/or death rate, but the stent in that study was still placed in the main trunk, such as ICA, M1, VA, and BA.^[32]^ Although there is no reliable data on surgical bypass, either direct or indirect bypass, to provide recommendations, bypass surgery has yielded positive outcomes in some studies.

Several surgical options, including direct, indirect, or combined bypass techniques, are available for revascularization to treat intracranial large-vessel occlusion.^[33–35]^ Direct revascularization comprises direct anastomosis of an external carotid artery branch (especially the Superficial temporal artery), acting as a donor, and a branch of the MCA, acting as a recipient. In the indirect technique, a vascularized tissue pedicle (temporalis muscle, dura, or artery with its adventitia) is placed on the brain surface, such as encephaloduroarteriomyosynangiosis and encephaloduroarteriosynangiosis. Another well-known indirect technique involves the use of multiple burr holes. Combined procedures involving direct and indirect techniques may also be performed. The choice of the optimal revascularization technique should be individualized for each patient. Surgery is performed when early clinical or radiologic signs of vasculopathy progression are observed, such as evidence of cerebral hypoperfusion, progressive narrowing of cerebral vessels, or the onset of relevant symptoms (e.g., headache, dysesthesia, and memory loss).

In our study, this patient was relatively young and possessed fewer CVA risk factors, such as old age, diabetes, hypertension, obesity, and hyperlipidemia. The strategy adopted shifted from maximizing medication to bypass surgery. Low et al reported that STA-M4 bypass surgery in carefully selected patients (impaired cerebral vasodilatory reserve) with intracranial ICA or MCA steno-occlusive disease resulted in significant improvement in hemodynamic parameters and reduction in stroke recurrence during a mean follow-up of 34 months.^[36]^ These studies had longer follow-up times than the median survival of GBM patients. In the absence of contraindications, antiplatelet or anticoagulation agents could have been added, and bypass surgery could be performed because there was no stent in the distal intracranial arteries. To our knowledge, this is the first report on bypass surgery in GBM patients with ORV.

## 4. Conclusion

MRA is a potential screening tool for ORV in GBM patients and that more attention should be paid to the vessels contained by the PTV. ORV should be suspected as an etiology of stroke in the young GBM population without the risk of atherosclerosis. Antiplatelet or anticoagulation agents can be added, and bypass surgery could be performed to improve brain perfusion.

## Author contributions

**Conceptualization:** Yang Chien-Tung.

**Supervision:** Chun-Chung Chen.

**Writing – original draft:** Yang Chien-Tung.

**Writing – review & editing:** Chun-Chung Chen.
